# Supplementary material for: Understanding the association between parental attitudes and the practice of female genital mutilation among daughters
Source: PLoS One. 2020 May 21;15(5):e0233344. doi: 10.1371/journal.pone.0233344 (PMC7241784; doi:10.1371/journal.pone.0233344)
Supplement: S1 Table — (DOCX) [file pone.0233344.s001.docx]

**Table S1. Percentage distribution of couples with at least one living daughter aged 0 to 14 years, by whether they have concordant or discordant opinions about the continuation of the practice**

|  | **Concordant** | | | | **Discordant** | | | | | |  |
| --- | --- | --- | --- | --- | --- | --- | --- | --- | --- | --- | --- |
|  | **Total** | **Both want FGM to continue** | **Both want FGM to end** | **Both undecided** | **Total** | **Woman wants FGM to continue, but man wants FGM to stop or is undecided** | **Man wants FGM to continue, but woman wants FGM to stop or is undecided** | **Woman wants FGM to stop, but man is undecided** | **Man wants FGM to stop, but woman is undecided** | **Other/missing** | **Number of couples with at least one living daughter aged**  **0 to 14 years** |
| Cameroon | 66.4 | 3.8 | 60.5 | 2.0 | 32.3 | 4.7 | 2.9 | 16.4 | 8.2 | 1.3 | 230 |
|  | (62.6-70.2) | (1.8-5.9) | (56.4-64.7) | (0.9-3.0) | (28.4-36.2) | (2.9-6.4) | (0.8-5.1) | (12.3-20.6) | (5.5-10.9) | (0.1-2.6) |  |
|  |  |  |  |  |  |  |  |  |  |  |  |
| Niger | 77.6 | 0.3 | 77.0 | 0.3 | 21.6 | 4.4 | 3.0 | 4.0 | 10.2 | 0.8 | 675 |
|  | (73.5-81.7) | (0-0.7) | (72.9-81.2) | (0-0.8) | (17.5-25.6) | (2.4-6.5) | (0.9-5.1) | (2.0-6.0) | (7.2-13.2) | (0.1-1.5) |  |
|  |  |  |  |  |  |  |  |  |  |  |  |
| Togo | 89.5 | 0.2 | 89.3 | 0.1 | 9.3 | 0.8 | 1.2 | 3.8 | 3.4 | 1.1 | 913 |
|  | (87.3-91.7) | (0-0.5) | (87.1-91.5) | (0-0.2) | (7.2-11.5) | (0.3-1.4) | (0.5-2.0) | (2.4-5.2) | (2.1-4.8) | (0.3-2) |  |
|  |  |  |  |  |  |  |  |  |  |  |  |
| Benin | 83.3 | 0.1 | 82.4 | 0.8 | 16.7 | 1.1 | 2.7 | 6.7 | 6.2 | 0.0 | 1200 |
|  | (80.7-85.8) | (0-0.3) | (79.6-85.2) | (0.2-1.4) | (14.2-19.3) | (0.3-1.8) | (1.6-3.8) | (5.0-8.5) | (4.8-7.7) |  |  |
|  |  |  |  |  |  |  |  |  |  |  |  |
| Tanzania | 83.0 | 0.4 | 82.6 | 0.0 | 17.0 | 4.5 | 6.0 | 3.3 | 3.1 | 0.0 | 521 |
|  | (80.0-85.9) | (0-1.0) | (79.7-85.5) |  | (14.1-20.0) | (2.8-6.3) | (4.1-8.0) | (1.7-5.0) | (1.7-5.6) |  |  |
|  |  |  |  |  |  |  |  |  |  |  |  |
| Kenya | 86.2 | 3.6 | 82.5 | 0.1 | 13.6 | 2.4 | 7.9 | 2.5 | 0.7 | 0.3 | 3289 |
|  | (84.8-87.6) | (2.8-4.4) | (80.8-84.2) | (0-0.1) | (12.1-15.0) | (1.9-2.9) | (6.7-9.1) | (1.9-3.2) | (0.5-1.0) | (0.1-0.4) |  |
|  |  |  |  |  |  |  |  |  |  |  |  |
| Senegal | 73.9 | 9.1 | 64.4 | 0.3 | 26.1 | 11.2 | 7.0 | 5.9 | 2.1 | 0.0 | 1493 |
|  | (71.0-76.8) | (7.1-11.2) | (60.7-68.2) | (0-0.6) | (23.2-29.0) | (9.1-13.2) | (4.9-9.1) | (4.4-7.3) | (1.3-2.9) |  |  |
|  |  |  |  |  |  |  |  |  |  |  |  |
| Nigeria | 50.3 | 9.1 | 39.8 | 1.3 | 48.8 | 13.6 | 20.0 | 6.4 | 8.9 | 0.9 | 3200 |
|  | (47.9-52.7) | (7.6-10.7) | (37.5-42.1) | (0.8-1.8) | (46.4-51.3) | (11.4-15.7) | (17.8-22.2) | (5.1-7.7) | (7.5-10.3) | (0.5-1.3) |  |
| Côte d’Ivoire | 72.4 | 6.7 | 65.5 | 0.2 | 27.0 | 11.2 | 9.0 | 4.1 | 2.8 | 0.5 | 1113 |
|  | (69.3-75.6) | (4.5-8.8) | (61.9-69.2) | (0-0.6) | (23.9-30.2) | (8.7-13.6) | (6.5-11.4) | (2.5-5.7) | (1.7-4.0) | (0.1-1.0) |  |
|  |  |  |  |  |  |  |  |  |  |  |  |
| Chad | 55.6 | 32.8 | 21.4 | 1.3 | 43.7 | 20.4 | 12.9 | 2.1 | 8.4 | 0.6 | 561 |
|  | (48.2-63.1) | (26.4-39.3) | (15.1-27.7) | (1.0-1.7) | (36.4-51.1) | (14.9-25.9) | (9.6-16.1) | (0.5-3.6) | (4.2-12.6) | (0-1.3) |  |
|  |  |  |  |  |  |  |  |  |  |  |  |
| Ethiopia | 69.1 | 4.3 | 64.8 | 0.0 | 30.9 | 17.4 | 9.0 | 1.6 | 2.9 | 0.0 | 2271 |
|  | (65.6-72.6) | (2.8-5.8) | (61.0-68.6) | (0-0.1) | (27.4-34.4) | (14.3-20.5) | (7.1-10.9) | (0.7-2.4) | (1.9-4.0) |  |  |
|  |  |  |  |  |  |  |  |  |  |  |  |
| Burkina Faso | 78.8 | 1.6 | 77.2 | 0.0 | 21.0 | 8.5 | 9.6 | 2.2 | 0.6 | 0.2 | 3489 |
|  | (76.7-80.9) | (0.8-2.5) | (74.7-79.6) |  | (18.9-23.1) | (7.2-9.8) | (7.9-11.3) | (1.6-2.9) | (0.3-0.9) | (0-0.4) |  |
|  |  |  |  |  |  |  |  |  |  |  |  |
| Sierra Leone | 50.2 | 42.3 | 7.0 | 0.9 | 49.3 | 37.5 | 8.3 | 1.7 | 1.9 | 0.5 | 2226 |
|  | (46.7-53.7) | (38.6-46.1) | (5.6-8.4) | (0.3-1.5) | (45.9-52.8) | (33.9-41.0) | (6.6-9.9) | (1.0-2.5) | (1.1-2.6) | (0.2-0.8) |  |
|  |  |  |  |  |  |  |  |  |  |  |  |
| Mali | 65.5 | 62.1 | 2.8 | 0.6 | 34.5 | 11.2 | 20.0 | 2.3 | 1.0 | 0.0 | 2144 |
|  | (62.6-68.4) | (59-65.3) | (1.7-3.9) | (0.3-0.9) | (31.6-37.4) | (9.3-13.2) | (17.5-22.5) | (1.4-3.1) | (0.5-1.5) |  |  |
| Guinea | 57.7 | 48.1 | 9.1 | 0.5 | 42.3 | 21.5 | 16.6 | 1.5 | 2.8 | 0.0 | 1634 |
|  | (54.4-61.0) | (44.6-51.6) | (7.2-11.0) | (0.2-0.8) | (39.0-45.6) | (18.5-24.4) | (13.8-19.3) | (0.7-2.4) | (1.7-3.8) |  |  |
|  |  |  |  |  |  |  |  |  |  |  |  |

Notes: Due to rounding, rows will not add exactly to 100%. The “other” category includes couples for whom the opinion of one partner is missing, while the “missing” category includes couples for whom the opinions of both partners are missing. For Chad, information on girls’ biological fathers was not collected, so couples shown in this table include girls’ mothers and their partners, who may not be the biological fathers in all cases.
