# Supplementary material for: Understanding the association between parental attitudes and the practice of female genital mutilation among daughters
Source: PLoS One. 2020 May 21;15(5):e0233344. doi: 10.1371/journal.pone.0233344 (PMC7241784; doi:10.1371/journal.pone.0233344)
Supplement: S2 Table — (DOCX) [file pone.0233344.s002.docx]

**Table S2. Percentage of daughters who have undergone FGM, by parental opinions about the continuation of the practice**

|  | **Concordant** | | **Discordant** | | | | **Number of daughters aged 0-14** |
| --- | --- | --- | --- | --- | --- | --- | --- |
|  | **Both want FGM to continue** | **Both want FGM to end** | **Woman wants FGM to continue, but man wants FGM to stop or is undecided** | **Man wants FGM to continue, but woman wants FGM to stop or is undecided** | **Woman wants FGM to stop, but man is undecided** | **Man wants FGM to stop, but woman is undecided** |  |
| **Mid-prevalence countries** | | | | | | | |
| Kenya | 24.6 | 0.8 | 7.1 | 5.9 | 2.4 | 5.2 | 5498 |
|  | (18.1-31.2) | (0.5-1.2) | (2.9-11.2) | (2.4-9.4) | (0-5.7) | (0-12.5) |  |
| p<0.0001 |  |  |  |  |  |  |  |
| Senegal | 74.6 | 1.4 | 45.4 | 16.1 | 6.0 | 6.8 | 2629 |
|  | (68.7-80.5) | (0.7-2.0) | (37.1-53.7) | (8.6-23.6) | (0.9-11.1) | (0-13.6) |  |
| p<0.0001 |  |  |  |  |  |  |  |
| Nigeria | 62.3 | 7.2 | 61.9 | 15.9 | 10.8 | 16.9 | 5587 |
|  | (55.1-69.5) | (5.3-9.1) | (54.3-69.5) | (11.8-19.9) | (4.7-16.8) | (10.1-23.7) |  |
| p<0.0001 |  |  |  |  |  |  |  |
| Côte d’Ivoire | 34.2 | 5.9 | 32.1 | 10.4 | 2.9 | 5.6 | 1724 |
|  | (19.4-49.1) | (3.4-8.4) | (23.5-40.7) | (4.5-16.4) | (0-6.9) | (0.5-10.7) |  |
| p<0.0001 |  |  |  |  |  |  |  |
| Chad | 22.7 | 2.0 | 18.0 | 11.9 | * | (10.7) | 561 |
|  | (16.6-28.9) | (0-4.7) | (8.1-27.9) | (5.6-18.2) |  | (0-29.5) |  |
| p<0.0001 |  |  |  |  |  |  |  |
| **High-prevalence countries** | | | | | | | |
| Ethiopia | 44.2 | 11.7 | 29.9 | 19.1 | 23.3 | 24.3 | 4076 |
|  | (27.1-61.3) | (8.9-14.5) | (22.3-37.4) | (11.5-26.7) | (1.6-45.0) | (10.3-38.3) |  |
| p<0.0001 |  |  |  |  |  |  |  |
| Burkina Faso | 55.2 | 8.2 | 51.4 | 7.4 | 10.2 | 41.2 | 5902 |
|  | (30.7-79.7) | (7.0-9.4) | (44.3-58.4) | (4.3-10.6) | (1.3-19.2) | (19.3-63.1) |  |
| p<0.0001 |  |  |  |  |  |  |  |
| Sierra Leone | 16.0 | 8.9 | 14.7 | 12.9 | (0.0) | (16.3) | 2323 |
|  | (13.3-18.7) | (4.4-13.4) | (11.2-18.2) | (7.7-18.0) |  | (4.1-28.5) |  |
| p<0.0001 |  |  |  |  |  |  |  |
| Mali | 77.5 | 16.2 | 74.1 | 41.6 | 31.7 | (45.3) | 3842 |
|  | (74.4-80.6) | (4.7-27.6) | (68-80.2) | (34.8-48.3) | (15.5-47.9) | (20.8-69.8) |  |
| p<0.0001 |  |  |  |  |  |  |  |
| Guinea | 41.9 | 10.2 | 34.0 | 24.0 | (46.8) | (24.4) | 2648 |
|  | (38.1-45.7) | (5.6-14.8) | (28.7-39.2) | (18.6-29.3) | (16.5-77.1) | (10.4-38.3) |  |
| p<0.0001 |  |  |  |  |  |  |  |

Notes: Estimates between parentheses are based on 25 to 49 unweighted cases. Asterisks indicate estimates that were suppressed because they are based on fewer than 25 unweighted cases. For Chad, and Sierra Leone, the denominators refer to the number of couples with at least one living daughter aged 0 to 14 years, and the data refer to the percentage of couples with at least one living daughter who has undergone FGM. Values are lower than those shown in Table 1, which reflect daughters of any age who have undergone FGM. For Chad, information on girls’ biological fathers was not collected, so couples shown in this table include girls’ mothers and their partners, who may not be the biological fathers in all cases.
