# Supplementary material for: Understanding the association between parental attitudes and the practice of female genital mutilation among daughters
Source: PLoS One. 2020 May 21;15(5):e0233344. doi: 10.1371/journal.pone.0233344 (PMC7241784; doi:10.1371/journal.pone.0233344)
Supplement: Attachment — Submitted filename: Response to reviewers.docx [file pone.0233344.s003.docx]

Claudia Cappa
Senior Adviser, Statistics
Data and Analytics Section
Division of Data, Analytics, Planning and Monitoring
UNICEF, 3 UN Plaza, New York, NY 10017

March 18, 2020

Dear Editor in Chief,

We thank the reviewers for their thoughtful and helpful comments. Specific comments are addressed below in red.

Sincerely,

Claudia Cappa, Claire Thomson, Colleen Murray

***

**Reviewer #1**

This is an interesting and valuable paper on analysis of DHS data from multiple countries on parental attitudes toward and support for FGM. While the analysis is sound, I found that both the introduction to the paper and the (lack of) discussion at the end limit its value to the field. Overall, I felt that the authors needed to provide a more detailed summary of recent published research on FGM, of which there is a considerable quantity including from the countries of interest in this study and including evidence of effective intervention strategies.

The section with the literature review has been revised significantly to add more detailed summary of recently published research on FGM.

Also, there is no discussion section. The authors state conclusions, but these are really just a restatement of the results section. There is no interpretation of findings, discussion of potential future research, or detail on future interventions or approaches that might be implied by this research. More detail needs to be provided on why this study matters to the field. The analysis is sound, but it's not clear why we should care. What does this study really tell us, and where does it suggest we should go from here?

A discussion section was added.

**Reviewer #2**

This article analyses the relationship between FGM and parental attitudes towards FGM in African countries where FGM is more or less common. The most interesting, if somewhat depressing, result is that mother's attitude trumps fathers attitude when they disagree. I find the paper convincing, well thought through and well written, though with substantial shortcomings that need to be addressed before I can recommend an accept.

1. Type of FGM is not included in the analyses. This could make a huge difference as some earlier studies have revealed a higher commitment to more costly rituals. Conversely, it may be easier to maintain a less costly commitment. Either way, type of FGM should be included in the analyses.

We did look at data on the circumstances surrounding the practice, including girl’s age at cutting, practitioner and type of cutting. The original intent was to see whether such circumstances would show differentials by parental attitudes. The hypothesis there was that, for instance, among couples with discordant opinions, the cutting may still happen but in milder forms or at younger age or with the involvement of a health professional as opposed to a traditional circumciser.

We later decided not to show the results in the paper for a number of reasons. Circumstances do not seem to change drastically by parental attitudes. Most daughters underwent FGM when they were 0 to 4 years old and in none of the countries did age at cutting significantly change by parents’ opinions. The majority of girls were cut by traditional practitioners regardless of parental opinions towards the practice. Finally, there was a certain level of variations across the countries in terms of types of FGM covered in the surveys. The only category that was constant was infibulation, which was rare in the countries with data. Finally, due to the low number of cut daughters in some countries, the analyses on age, practitioner and type of FGM by parental attitudes had a very large level of uncertainly and many values had to be suppressed, which made the results less reliable, and therefore not that meaningful.

2. These results can be much more transparently presented in graph format. It is unnecessarily difficult to go through all results in table form. Please rectify this.

Two graphs were added to illustrate the findings in tables 2 and 3.

3. Where are the results from the statistical tests? It is stated in the text that there should be tests included, but these results are nowhere to be found. Thus, I don't really know if the results are what the authors say they are or not.

The statement in the article was rectified to reflect only the actual type of analyses that were performed.

Additional requirements:

When submitting your revision, we need you to address these additional requirements:

1. Please ensure that your manuscript meets PLOS ONE's style requirements, including those for file naming. The PLOS ONE style templates can be found at <http://www.plosone.org/attachments/PLOSOne_formatting_sample_main_body.pdf> and <http://www.plosone.org/attachments/PLOSOne_formatting_sample_title_authors_affiliations.pdf>

 Style requirements were reviewed and changed were made to reflect the requirements.

2. Please consider revising your Abstract and make sure it it structured in terms of clearly defined background, objective, methods, result, conclusions. Refer to our submission guidelines for further guidance (<https://journals.plos.org/plosone/s/submission-guidelines#loc-abstract>). In addition, please refrain from stating p values as 0.000, either report the exact value or use the format p<0.0001.

 The abstract was revised to follow the suggested structure. P values were modified.

3. PLOS requires an ORCID iD for the corresponding author in Editorial Manager on papers submitted after December 6th, 2016. Please ensure that you have an ORCID iD and that it is validated in Editorial Manager. To do this, go to ‘Update my Information’ (in the upper left-hand corner of the main menu), and click on the Fetch/Validate link next to the ORCID field. This will take you to the ORCID site and allow you to create a new iD or authenticate a pre-existing iD in Editorial Manager. Please see the following video for instructions on linking an ORCID iD to your Editorial Manager account: <https://www.youtube.com/watch?v=_xcclfuvtxQ>

An ORCID was created and validated.

4. Please amend your manuscript to include your abstract after the title page.

The abstract was added after the title page.
