# Supplementary material for: Understanding the association between parental attitudes and the practice of female genital mutilation among daughters
Source: PLoS One. 2020 May 21;15(5):e0233344. doi: 10.1371/journal.pone.0233344 (PMC7241784; doi:10.1371/journal.pone.0233344)
Supplement: Attachment — Submitted filename: Response to reviewers - 1 May.docx [file pone.0233344.s004.docx]

Claudia Cappa
Senior Adviser, Statistics
Data and Analytics Section
Division of Data, Analytics, Planning and Monitoring
UNICEF, 3 UN Plaza, New York, NY 10017

May 1, 2020

Dear Editor in Chief,

We thank you for the quick feedback and helpful comments. Our responses are available below in red.

Additionally, we have done a careful editing and factchecking of the paper, which explains the additional track changes.

Sincerely,

Claudia Cappa, Claire Thomson, Colleen Murray

***

1. In your response to Reviewer 2, you give reasons for not distinguishing between different types of cutting. I accept these reasons. However, in the method section of the manuscript you need to report what types of cutting are included in the FGM measure you report, and you need to briefly state why different types are treated as a single category in your analysis.

A sentence was added to describe the type of FGM included in the analysis.

2. In the Figure 1 note, I think the sentence "Due to rounding, values will not add exactly to 100%" should be removed (it only applies to the table, right?).

The sentence above was removed.

3. In the Figure 1 note, add a sentence about the accuracy of these percentages, e.g., "The width of 95% confidence intervals for these percentages were typically just a few percentage points, see Table S1."

Confidence intervals were added to Figure 1.

4. In Figure 2, confidence intervals should be included as error bars in the bar diagram.

Confidence intervals were added to Figure 2.

5. State explicitly which null hypothesis the p-values refer to. My guess is that the null hypothesis (that you reject in all countries except one) is "the prevalence of FGM does not depend on parents' opinions".

The null hypothesis was made clear in the section on methods and the main finding in terms of statistical association was emphasized in the section on results.
